# Worm-Based Microfluidic Biosensor for Real-Time Assessment of the Metastatic Status

**DOI:** 10.3390/cancers13040873

**Published:** 2021-02-19

**Authors:** Jing Zhang, Song Lin Chua, Bee Luan Khoo

**Affiliations:** 1 Department of Biomedical Engineering, City University of Hong Kong, Hong Kong, China; jingzha22-c@my.cityu.edu.hk; 2 Department of Applied Biology and Chemical Technology, The Hong Kong Polytechnic University, Hong Kong, China; song-lin.chua@polyu.edu.hk; 3 State Key Laboratory of Chemical Biology and Drug Discovery, The Hong Kong Polytechnic University, Kowloon, Hong Kong, China; 4 Shenzhen Key Laboratory of Food Biological Safety Control, Shenzhen 518000, China

**Keywords:** metastasis, disease monitoring, worm-based biosensor, preclinical models, label-free

## Abstract

**Simple Summary:**

We proposed a high-throughput screening and low-cost worm-based (WB) microfluidic biosensor to monitor biochemical cues related to metastasis. *Caenorhabditis elegans* placed in the WB biosensor chambers and exposed to samples conditioned with cancer cell clusters reflect differences in the chemotactic preference of worms. We observed a higher distribution of worms associated with samples of higher metastatic potential (*p* < 0.005). A chemotaxis index (CI) was defined to standardize the quantitative assessment from the WB biosensor, where increased metastatic potential was associated with higher CI levels (6.5 ± 1.37). We found that the secreted metabolite glutamate was a chemorepellent, and lower glutamate levels were associated with samples derived from more metastatic cancer cell clusters. In conclusion, WB biosensors could evaluate patient status in real time, thereby facilitating early detection of metastases and routine management.

**Abstract:**

Background: Metastasis is a complex process that affects patient treatment and survival. To routinely monitor cancer plasticity and guide treatment strategies, it is highly desired to provide information about metastatic status in real-time. Here, we proposed a worm-based (WB) microfluidic biosensor to rapidly monitor biochemical cues related to metastasis in a well-defined environment. Compared to conventional biomarker-based methods, the WB biosensor allowed high throughput screening under low cost, requiring only visual quantification of outputs; Methods: *Caenorhabditis elegans* were placed in the WB biosensor and exposed to samples conditioned with cancer cell clusters. The chemotactic preference of these worms was observed under discontinuous imaging to minimize the impact on physiological activity; Results: A chemotaxis index (CI) was defined to standardize the quantitative assessment from the WB biosensor, where moderate (3.24–6.5) and high (>6.5) CI levels reflected increased metastasis risk and presence of metastasis, respectively. We demonstrated that the secreted metabolite glutamate was a chemorepellent, and larger clusters associated with increased metastatic potential also enhanced CI levels; Conclusions: Overall, this study provided a proof of concept for the WB biosensors in assessing metastasis status, with the potential to evaluate patient-derived cancer clusters for routine management.

## 1. Introduction

The process of cancer spreading to other organs is termed metastasis [1]. The onset of metastasis is the leading cause of death in cancer patients [2]. In particular, breast cancer is currently the most common cancer worldwide and the leading cause of cancer-related mortality [2]. Blood-based tumor markers are a potential alternative for the non-invasive detection of cancer. For example, serum carcinoembryonic antigen is often used to assess the prognosis of cervical patients [3]. MicroRNAs have also been identified as a potential diagnostic and prognostic factor [4]. However, only a few studies have directly validated the clinical significance of these biomarkers [5]. Other markers correlated with metastatic risk have also been reported, but the incidence of false-positive and false-negative results is high [6]. Therefore, it is pivotal to quickly identify new tools that can rapidly screen and clinically validate cancer prognosis complementary to existing staging systems such as the T (tumor) N (node) M (metastasis) staging system [7].

*Caenorhabditis elegans* live in temperate soil environments and are transparent, with a length of about 1 mm [8,9]. *C. elegans* are hermaphrodites and can be easily handled in the laboratory. Therefore, it is widely used as an animal model in behavioral dynamics [10], neuronal imaging [11], and microsurgery applications [12,13,14]. The multisensory perception of *C. elegans* allows the worms to detect various volatile or water-soluble substances in food or other animals at low concentrations and avoid danger through a highly developed chemosensory system [15]. Compared with standards obtained from healthy cell cultures, *C. elegans* showed a chemotactic preference for specific volatile compounds in cells, urine, and tissue samples of cancer patients [16]. The detection of volatile compounds was attributed to olfactory neurons, as evidenced by nematode odor detection tests involving odr-3 mutant worms [16,17].

There are currently no studies evaluating the chemotactic preference of *C. elegans* for specific cancer subtypes. In vitro models are invaluable tools for high throughput screening and cancer biomarkers detection for prognosis and routine monitoring. Here, we developed a worm-based (WB) biosensor to monitor biochemical cues related to metastasis. The WB biosensor could reflect differences in the chemotactic preference of worms for samples obtained from cancer cell clusters of higher metastatic potential. The chemotaxis index (CI) was derived to quantify readouts from the WB biosensor. Glutamate is a component of glutamine metabolism in cancer cells associated with a malignant phenotype [18,19] and is reported to be secreted by breast cancer cells at high concentrations [20]. We demonstrated that the metabolite glutamate secreted from cancer cells served as a chemorepellent, and lower glutamate levels were associated with samples derived from more metastatic cancer cell clusters. When glutamate was degraded by glutamate dehydrogenase, the positive CI level readout for samples derived from more metastatic cancer cell clusters was abolished. Larger clusters associated with increased metastatic potential could also enhance CI levels.

To the best of our knowledge, this is the first pilot study that demonstrated the application of WB biosensors in the detection of metastatic cancer phenotypes. The WB biosensor can be potentially used to routinely screen patient-derived cultures from a liquid biopsy or tumor biopsy for early detection of metastasis [21]. These results will pave the way for biomarker discovery or the use of WB biosensors to monitor patient status in real-time, thereby facilitating the early detection of metastases.

## 2. Materials and Methods

### 2.1. WB Biosensor Operations

#### 2.1.1. Device Fabrication

**Figure 1:**
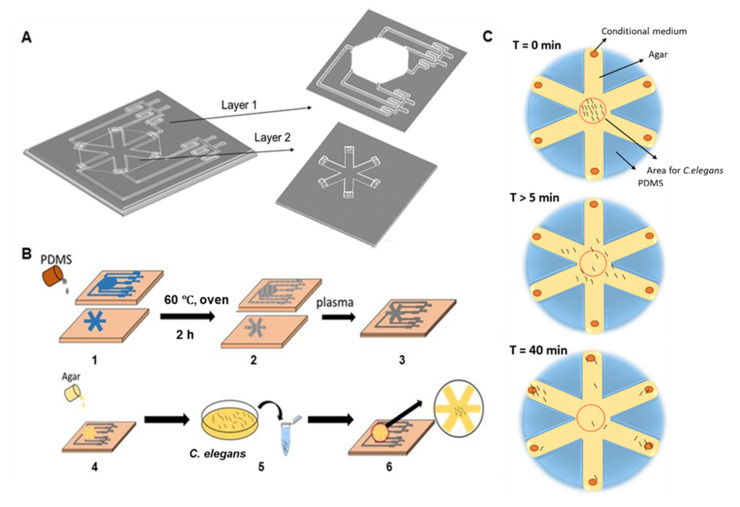
Schematics of the worm-based (WB) biosensor for the detection of metastatic disease. (**A**) Overview of the assay design for the WB biosensor. Worms were placed into chambers connected to inlets for sample loading. (**B**) The workflow of device fabrication and biosensor operations. (1) PDMS was poured into the device mold and cured at 60 °C for 2 h. (2) Resultant device layers. (3) Device layers were bonded by standard plasma treatment. (4) 1.5% agar solution was coated on the channels. (5) *C. elegans* were harvested in a 1.5 mL tube. (6) *C. elegans* were added to the WB biosensor. Imaging was done in situ with phase-contrast microscopy. (**C**) Experimental procedures with the WB biosensor. PDMS = polydimethylsiloxane; T = time.

Microfluidic chips were fabricated using standard soft-lithography techniques in PDMS described elsewhere (SYLGARD™ 184 silicone elastomer kit) [21]. First, we used the designed molds (length: 25 mm, width: 10 mm, height: 3 mm, loading area: 150 mm^2^) to fabricate the WB biosensor (Figure 1A,B). Each device consisted of two layers and had the following components: (1) interconnected channels with four sample inlets to investigate the influence of the biochemical cues on the chemotactic preference of *C. elegans*; (2) two gradient generators to establish a range of sample concentrations; (3) agar-coated loading chambers for the migration of *C. elegans* (Figure 1A). Each connecting channel was 2 mm × 2 mm (width × height). After the fabrication of individual biochip layers, the sealed biochip was obtained by manual alignment and oxygen plasma bonding. The respective fluidic inlets and outlets were punched into the layers before bonding. The WB biosensor channels could be primed within 1 min at a flow rate of 7.7 mL/min, leading to the formation of droplets (~10 µL) at each outlet of the loading chamber.

#### 2.1.2. *C. elegans* Loading

The 1.5% agar-coated loading chambers (150 mm^2^) (Sigma, St. Louis, Germany) were designed to hold the *C. elegans* before migration (Figure 1A). Adult *C. elegans* were collected in 1.5 mL tubes. About 50 L4-stage animals were used per experiment after washing twice with phosphate buffer saline (PBS) buffer (Gibco, Waltham, MA, USA) before the experiment. The worms initiated migration within 5 min after loading, and the distribution of worms was quantified 40 min after sample exposure. The quantification of worms was done by imaging in situ with phase-contrast microscopy.

#### 2.1.3. Sample Collection from Cancer Cell Cluster Cultures

Samples were obtained from cancer cell clusters after 48 h, maintained in microfluidic chips that were fabricated using standard soft-lithography techniques in PDMS described elsewhere [21]. The microfluidic device was composed of two layers: the barrier layer and the microwell layers. A barrier layer was a PDMS layer produced from a 3D printed master mold, which separated each microwells column into individual channels. The microwells layer containing cell clusters was produced from mold by standard photolithography [21]. The two layers were bonded together by oxygen plasma bonding.

### 2.2. C. elegans Culture

*C. elegans* were obtained from the Hong Kong Poly-University and cultured in the OP50 (a strain of *Escherichia coli* used to maintain *C. elegans* cultures) + nematode growth media plate (Biofil, Guangzhou, China). The experiments were carried out at room temperature for 40 min. The distribution of worms was quantified using phase-contrast microscopy.

For viability assessment, motility was assessed by probing with an inoculating needle. Worms were considered non-viable following no movement with probing stimuli.

### 2.3. Cell Culture

Breast cancer cell lines representing less metastatic (MCF-7) and more metastatic (MDA-MB-231) subtypes, as well as healthy fibroblast cells (HTB-22, HTB-26, Primary Tay-Sachs American Type Culture Collection), were used and maintained in a T25 flask (Biofil, Guangzhou, China) at 37 °C in 5% CO2 in Dulbecco’s modified Eagle’s media (DMEM) (Gibco, USA) supplemented with 10% FBS (Gibco, Waltham, MA, USA), 1% penicillin-streptomycin (Gibco). Cells were enumerated and seeded separately in different channels of the microfluidic device. The cell media were changed every 2–3 days, and cells were harvested when their confluence reached 80%.

For the microwell assay, conditional media was cultured for 48 h from MCF-7 and MDA-MB-231 cancer cells. The media was collected from cultures after 48 h and centrifuged to remove cellular debris.

### 2.4. Screening of Samples with the WB Biosensor

The conditional media were diluted at various factors (10^−1^, 10^−2^, and 10^−3^) with PBS buffer and added to the loading chamber. PBS buffer and samples from healthy cell cultures were used as standard references. Migration is initiated about 5 min after introduction to the loading chamber. The CI level was defined as stated in Equation (1):

CI = (percentage of *C. elegans* attracted to sample 1)/(percentage of *C. elegans* attracted to sample 2) (1)

The denatured samples from MCF-7 and MDA-MB-231 cancer cell clusters were prepared by heating the samples in a 60 °C oven for 30 min. Denatured samples were introduced to the WB biosensor at room temperature.

### 2.5. Glutamate Detection and Degradation Assays

Glutamate powder (Sigma, St. Louis, Germany) used in chemotaxis experiments was weighed and diluted to a range of concentrations with PBS buffer. A glutamate detection kit was used to evaluate the glutamate concentration levels in samples (Jian Cheng, A074, Nanjing, China). Samples were added to a 96-well plate (Corning, Corning, NY, USA), and OD values were analyzed by a microplate reader, as recommended by the manufacturer. Glutamate levels of samples were obtained with Equation (2):

glutamate level = ((sample OD 2 − sample OD 1) − (blank OD2 − blank OD 1))/ ((standard OD 2 − standard OD 1) − (blank OD 2 − blank OD 1)) × standard concentration × dilution ratio(2)

For glutamate degradation assays, the samples from MCF-7 and MDA-MB-231 cultures were incubated with glutamate dehydrogenase, recommended by the manufacturer (Jian Cheng, A074, Nanjing, China) in a ratio of 100:1 for 50 min. Samples were introduced to the WB biosensor at room temperature.

### 2.6. Statistical Analysis

All experiments were performed in triplicates. The results were presented as the mean ± standard deviation (SD). Groups were compared using a *t*-test (ANOVA) to evaluate associations between independent variables, and the *p*-values were obtained.

## 3. Results

### 3.1. Worm-Based (WB) Microfluidic Biosensor for the Detection of Metastasis

To routinely screen samples and evaluate the onset of metastasis under high throughput in a cost-effective manner, we developed a WB microfluidic biosensor, which consisted of two layers and had the following components: (1) Interconnected channels with four sample inlets to investigate the influence of the biochemical cues on the chemotactic preference of *C. elegans*; (2) Two gradient generators to establish a range of sample concentrations; (3) Agar-coated loading chambers for the migration of *C. elegans* (Figure 1A). The WB biosensor was fabricated with polydimethylsiloxane (PDMS) and sealed with a base layer by plasma treatment to allow loading and containment of samples (Figure 1B). The samples from cancer cell clusters were introduced through the inlets by a pump infusion system.

*C. elegans* has been well-established to detect volatile compounds or proteins [13,14,15] and was therefore utilized as our animal model. To initiate experiments, we first harvested the adult worms and placed them in the WB biosensor loading area (Figure 1C). The quantification of worms was done by imaging in situ with phase-contrast microscopy. The worms initiated migration within 5 min after loading, and the distribution of worms was quantified 40 min after sample exposure.

### 3.2. Characterization of Parameters for the WB Biosensor

**Figure 2:**
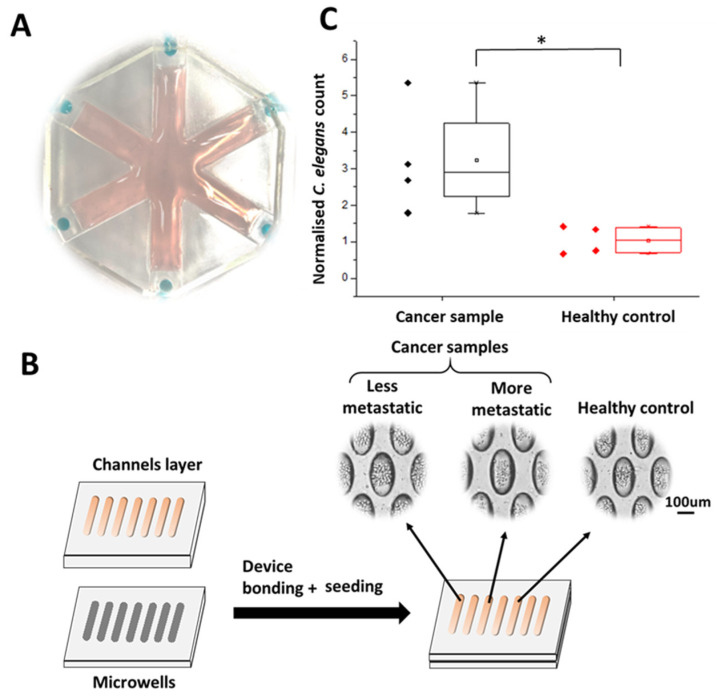
Characterization of the WB biosensor. (**A**) A representative image of the multiplexed WB biosensor for parallel screening. (**B**) Schematics of the device and workflow used to generate samples from cancer cell clusters within 48 h. (**C**) Box plot reflecting the chemotactic preference of worms with the WB biosensor for less metastatic cancer samples (MCF-7) and healthy controls (fibroblast). Worm counts were normalized to those obtained with PBS buffer standards. * states for *p* values < 0.05.

Due to the advantages of microfluidic technology, the design of the WB biosensor could be modified according to the number of samples screened. Here, we used a WB biosensor consisting of six loading chambers for parallel screening (Figure 2A). Samples were obtained from cancer cell microclusters maintained in a microwell-based array. The use of the microwell array allowed three-dimensional clusters of consistent composition to be generated [21]. Concentrated samples conditioned by cancer cell clusters (150 µL from 256 cell clusters) retained biochemical compounds that could serve as chemotactic cues (Figure 2B). Samples from the cancer cell clusters were collected after 48 h. Each cluster had approximately 50 cells (Figure 2B). The WB biosensor channels could be primed within 1 min at a flow rate of 7.7 mL/min, leading to the formation of droplets (~10 µL) at each outlet of the loading chamber.

We first evaluated the cell seeding concentrations required to demonstrate clinical correlations, using a range of cell seeding concentrations (1.5 × 10^4^, 2.5 × 10^4^, and 3.5 × 10^4^ cells per channel). The cancer cell clusters were prepared at a seeding concentration of 3.5 × 10^4^ per channel to achieve a range of diameter approximately 137.41 ± 29.69 µm, mimicking the presence of small micrometastasis (~0.2 mm) [22]. The proportion of cluster formation within this size range was also highest at the seeding concentration of 3.5 × 10^4^ (Figure S1). The resulting cancer cell clusters were under a negligible amount of shear, leading to high cellular viability (90.31 ± 5.9%) (Figure S2).

We aimed to validate the utility of the WB biosensor to distinguish cancer samples from healthy controls by evaluating the chemotactic preference of *C. elegans* to samples obtained from cancer cell clusters with lower metastatic potential, using MCF-7 cell lines (Figure 2C). There were no significant differences in terms of the viability of the *C. elegans* before and after experiments (40 min), as determined by their motility (>89%; Figure S3), implying that the cancer samples were not toxic to the animals. We confirmed that the distribution of *C. elegans* was higher in channels loaded with samples from cancer clusters, compared to that from healthy fibroblast controls (3-fold, *p*-value < 0.05) (Figure 2C). These observations were consistent with previous studies that suggested a chemotactic presence of *C. elegans* for cancer samples compared to healthy samples [16].

### 3.3. Detection of Samples Associated with Higher Metastatic Potential Using the WB Biosensor

Tumors exhibit intratumoral heterogeneity, promoting cancer progression, treatment failure, and disease recurrence [23]. Cancer cell plasticity is an important mechanism that generates a diversity of cancer cells, allowing cancer cells to alter their state and acquire plasticity in response to physiological stresses such as treatment selection and oncogenic stress [24]. There is a need to develop new tools for routine and low-cost evaluation of metastatic onset during treatment.

We hypothesized that higher metastatic potential cancer cells provide different biochemical cues that our WB biosensor could detect. To evaluate our hypothesis, we investigated the chemotactic preferences of *C. elegans* to samples from different cancer phenotypes. Breast cancer cell lines MCF-7 and MDA-MB-231 represented the less metastatic and more metastatic cancer phenotypes, respectively [25].

To standardize the quantitative readouts from the WB biosensor, we derived a chemotaxis index (CI), as stated in Equation (1).

**Figure 3:**
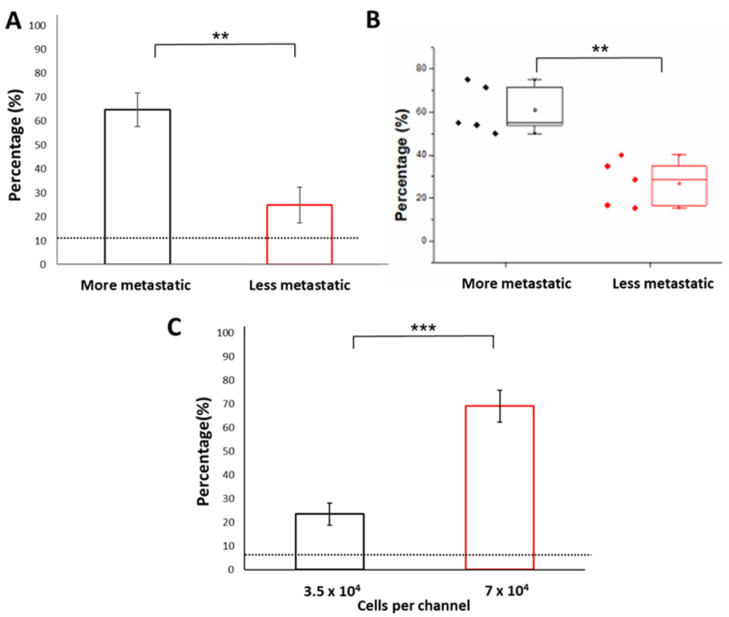
Detection of metastatic phenotypes with the WB biosensor. (**A**) Bar chart reflecting the chemotactic preference of worms to samples from more metastatic cancer cells (MDA-MB-231) compared to samples from less metastatic cancer cells (MCF-7). The dotted line corresponded to the proportion of *C. elegans* with samples from healthy controls (fibroblast). (**B**) The proportion of *C. elegans* when exposed to samples denatured by heat treatment. (**C**) The proportion of *C. elegans* when exposed to samples obtained from larger clusters of higher metastatic potential. The dotted line corresponded to the proportion of *C. elegans* with samples from PBS buffer standards. *** states for *p* < 0.0001, ** states for *p* < 0.005.

We first evaluated the chemotactic preference of cancer samples against standards obtained from either PBS buffer or healthy cell cultures. The average CI levels obtained between PBS (as sample 1) and buffer healthy standards (as sample 2) was 1.04 ± 0.39, reflecting a negligible difference in worm distribution. We previously demonstrated that the distribution of *C. elegans* was higher in channels loaded with samples obtained from cancer cell clusters with lower metastatic potential (MCF-7), compared to that of controls (3-fold, *p*-value < 0.05) (Figure 2C). However, the resultant CI level for samples obtained from cancer cell clusters of less metastatic potential was still lower (3.24 ± 1.52) than that obtained from samples obtained from cancer cell clusters of higher metastatic potential (MDA-MB-231) (6.5 ± 1.37; *p* < 0.005), in comparison to PBS buffer control (Figure 2C). The CI levels were obtained with samples of higher and lower metastatic potential, respectively (Figure 3A and Figure 2C). The CI levels reflected an overall outcome derived from various chemotactic agents present in the sample, including volatiles [16]. Specifically, compared with samples obtained from cancer cell clusters with lower metastatic potential (24.91 ± 7.51%), *C. elegans* had a significantly higher chemotactic preference for samples obtained from cancer cell clusters of higher metastatic potential (64.72 ± 6.98%, 2.6-fold) (Figure 3A). These observations confirmed that cancer cells of the higher metastatic potential provided different biochemical cues that our WB biosensor could detect.

Interestingly, the denaturation of samples by heat treatment did not abolish the chemotactic preference of *C. elegans* to samples of higher metastatic potential. The heat treatment was administered to determine if the properties of the chemotactic agent were retained under high temperature. The persistence of chemotactic preference of *C. elegans* to metastatic samples suggested that the chemotactic agent for metastasis was not volatile or protein-based (Figure 3B).

Clinically, larger tumors are associated with multifocal diseases [26]. The presence of multifocality is defined as two or more distinct and separate cancerous lesions in the breast, which is also related to the size of the tumor. Compared with single-focal breast cancer, multifocal breast cancer has a higher risk of vascular invasion and lymph node metastasis [27,28,29,30,31]. Larger tumor size is also associated with worsening patient prognosis [32]. Therefore, to mimic the presence of larger tumors associated with a higher risk of metastatic disease, we increased the cell seeding concentration (7 × 10^4^ cells per channel) to produce larger cell clusters (increase by 4-fold; 8383.96 ± 2373.2 µm^2^) (Figure S4). We successfully demonstrated that the WB biosensor could reflect the preference of *C. elegans* for samples from larger cancer cell clusters (3-fold, 69.11%; *p*-value: 0.00003) (Figure 3C). Therefore, the WB biosensor could be used for various sample types of increased metastatic potential.

### 3.4. Low Glutamate Levels Reflect the Presence of Metastasis

Glutamate is a component of glutamine metabolism in cancer cells associated with a malignant phenotype [18,19] and is reported to be secreted by breast cancer cells at high concentrations [20]. Therefore, we hypothesized that glutamate could be a chemotactic agent for *C. elegans* to detect various cancer subtypes.

**Figure 4:**
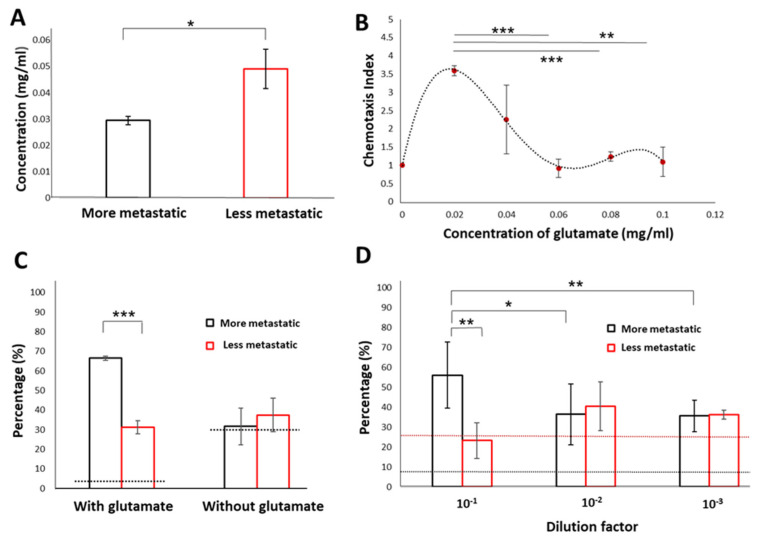
Glutamate acts as a chemorepellent to reflect the presence of metastatic phenotypes. (**A**) Glutamate levels in samples from cancer cells of the more metastatic phenotype (MDA-MB-231) and cancer cells of the less metastatic phenotype (MCF-7). (**B**) Quantitative evaluation of outcomes from the WB biosensor by establishing a chemotaxis index (CI). A range of glutamate concentrations (0.02 mg/mL, 0.04 mg/mL, 0.06 mg/mL, 0.08 mg/mL, and 0.1 mg/mL) was evaluated. (**C**) The proportion of *C. elegans* under exposure to samples from cancer cells of the more metastatic phenotype (MDA-MB-231) and cancer cells of the less metastatic phenotype (MCF-7) before and after glutamate degradation. The dotted line corresponded to the averaged value obtained with PBS buffer standards. (**D**) Evaluation of the detection limit for the WB biosensor. The black dotted line reflected the threshold determined by the rate of false-positives (8.71 ± 0.033%). The red dotted line reflected the threshold determined by the averaged value obtained with undiluted MCF-7 samples (25.73 ± 0.054%). *** states for *p* < 0.0005, ** states for *p* < 0.005 * states for *p* < 0.05.

We first quantified the glutamate levels from samples of cancer cell clusters with various metastatic potential. The glutamate levels from samples of cancer cell clusters with less metastatic potential (1.69-fold; MCF-7:0.049 ± 0.0074 mg/mL) were higher than that of cancer cell clusters with more metastatic potential (MDA-MB-231:0.029 ± 0.0015 mg/mL) (Figure 4A). These results support previous studies demonstrating a higher metabolite consumption and semiquantitative production of glutamate by 2D MCF-7 cell cultures [33].

We further evaluated the CI level readouts with the WB biosensor using a range of glutamate concentration levels that included concentrations detected from MCF-7 and MDA-MB-231 clusters (0.02 mg/mL, 0.04 mg/mL, 0.06 mg/mL, 0.08 mg/mL, and 0.1 mg/mL) (Figure 4A). CI level readouts were modified for parallel comparison of all concentrations, with the CI level obtained from PBS buffer standards defined as 1. We demonstrated that the glutamate concentration of 0.02 mg/mL generated the most significant CI level (3.6 ± 0.03), which was within the range of glutamate levels detected from cancer cell clusters with more metastatic potential (MDA-MB-231). Higher concentrations of glutamate (≥0.04 mg/mL) led to lower CI levels. Specifically, the lowest CI level was reported with the highest glutamate concentration tested (0.1 mg/mL; CI level: 1.1 ± 0.04) (Figure 4B).

We demonstrated that glutamate degradation by glutamate dehydrogenase could reduce CI level readouts with the WB biosensor to levels corresponding to samples from cancer cell clusters of higher metastatic potential, thus confirming the role of glutamate as a chemorepellent (Figure 4C). Moreover, other chemoattractants such as volatile compounds could still be present in cancer samples [16,34], which retained the chemotactic preference of *C. elegans* towards cancer samples over healthy controls, despite the degradation of glutamate.

We evaluated the detection limit of the WB biosensor with samples from both metastatic and less-metastatic cancer cell clusters under various dilutions (10^−1^, 10^−2^ and 10^−3^). The threshold was determined by the false positive rate (8.71 ± 3.33%) obtained from the proportion of *C. elegans* distributed to chambers loaded with samples from control groups. Chemotactic preference of *C. elegans* towards samples from metastatic cancer cell clusters was abolished at dilutions higher than 10^−1^ (Figure 4D). The dilution factor of 10^−2^ corresponded to an approximate glutamate concentration of 0.003 mg/mL.

## 4. Discussion

**Figure 5:**
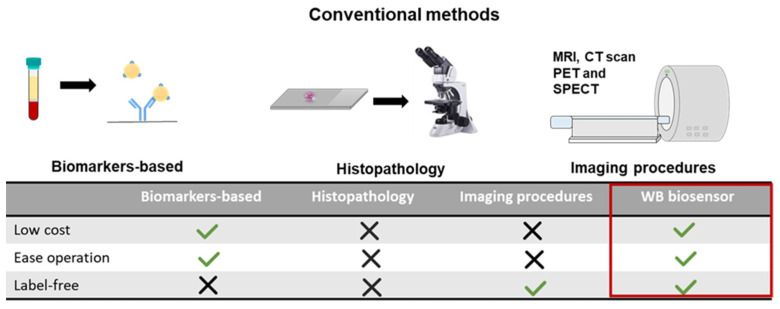
Advantages of the WB biosensor for metastatic detection. (Above) Schematics of conventional methods for metastatic detection. (Below) The low-cost, label-free, and ease of operation for the WB biosensor will promote widespread utility compared to techniques including biomarkers-based assays, histopathology, and imaging [22,35,47,48,49]. MRI = magnetic resonance imaging; CT = computed tomography scan; PET = positron emission tomography; SPECT = single-photon emission computer tomography.

Metastasis is a complex process in which primary tumor cells migrate and establish secondary tumors by invading specific tissues or disseminating blood and lymphatic systems [35]. Early detection of metastatic disease is of great significance for reducing mortality and improving overall survival to facilitate timely treatment and intervention. Existing metastatic detection methods usually require long-processing time, expensive equipment, and trained personnel for analysis and operations (Figure 5, Table S1). Current methods for detecting metastatic disease in the clinical setting include those that require the use of specific biomarkers in circulation (e.g., serum), secretions (e.g., urine), or tissue biopsies, such as cancer antigen 15–3 and carcinoembryonic antigen (Figure 5) [36,37]. Histopathology involves the microscopic examination of cells by a trained pathologist and requires an extended processing time [38]. Other techniques include imaging procedures such as magnetic resonance imaging (MRI), X-rays, radiography, computed tomography scan (CT scan), and nuclear imaging, e.g., positron emission tomography (PET) and single-photon emission computer tomography (SPECT) [39,40]. However, some cancers are almost invisible or hard to detect on a CT scan, such as prostate cancer, uterine cancer, and certain liver cancers. On MRI, bone and brain metastases are detected easier, and various cancer phenotypes can be distinguished based on localization. However, these imaging procedures are costly and have certain limitations, such as detecting lymph node micrometastases [41,42]. PET is another one of the most common approaches for clinical imaging techniques [43]. PET can detect recurrent disease and lymph node involvement at an early stage [44]. However, these imaging techniques require careful interpretation due to the possibility of observing age-related benign pathologies and the flare phenomenon associated with an increased uptake of radiotracer caused by hormone therapy [45]. Interpretations can also be affected by patient conditions such as blood glucose levels or psychotropic drugs [46].

Other methods focusing on detecting biomarkers demonstrate high specificity but low sensitivity due to the heterogeneous nature of cancer. Here, we showed that samples from cancer cell clusters of higher metastatic potential could be distinguished from samples of a lower metastatic potential using the WB biosensor. We envisioned that this label-free strategy for rapid screening at low cost and high throughput would allow timely identification of patients with potential metastases, complementing diagnostic techniques to direct samples of higher risk for further evaluation.

**Figure 6:**
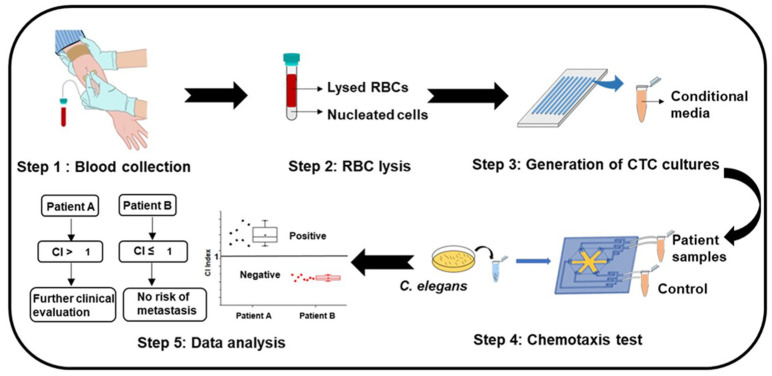
The proposed workflow of the WB biosensor for clinical utility. Step 1: collection of blood from patients. Step 2: preprocessing of samples to remove red blood cells through lysis. Step 3: short-term culture of nucleated cells under suitable conditions to generate CTC clusters [54]. Step 4: collection of samples from patient-derived cultures for rapid screening with the WB biosensor. Step 5: analysis of the chemotactic index (CI). A positive index (CI > 1) reflects the need for further evaluation, while a negative index reflects no metastasis risk. RBC = red blood cells; CTC = circulating tumor cell.

*C. elegans* have been widely reported to be chemotactic for specific odorants [16]. Here, we reported that *C. elegans* could also detect non-odorants, such as glutamate. Lower glutamate levels reflected the presence of samples from cancer cell clusters of higher metastatic potential. Further studies could be carried out to discover other potential chemotactic agents as biomarkers of metastasis. The cost-effective, portable, label-free, and ease of operations for the WB biosensor allows potential utility in a wide range of cancer subtypes for clinical significance and application prospects. For example, circulating tumor cells (CTCs) associated with overall patient survival and prognosis [50] could be extracted and concentrated from the liquid biopsy of patients using established techniques [51,52,53] for screening with the WB biosensor (Figure 6). Clinical samples would be compared against buffer standards to establish CI levels. Using the CI index, a readout of CI level from 3.24–6.5 would reflect a heightened risk of metastasis, while a CI level > 6.5 would reflect the presence of metastasis. Patient samples could also be compared against MCF-7 standards instead of PBS standards, where a CI index > 1 would reflect positivity and suggest further evaluation. We envisioned that the WB biosensor could complement routine screening of patients during treatment to evaluate the risk of metastatic onset, allowing timely referral for treatment intervention.

## 5. Conclusions

Here, we demonstrated the early detection of metastasis using a WB biosensor. A chemotaxis index (CI) was defined to reflect increased metastasis risk or presence of metastasis. We suggested that the presence of secreted metabolite glutamate acted as a chemorepellent and demonstrated that higher metastatic potential samples produced lower glutamate levels. Furthermore, glutamate degradation effectively abolished positive CI-level readouts obtained with samples from cancer cell clusters of higher metastatic potential. Larger clusters associated with increased metastatic potential also enhanced CI levels. Overall, the WB biosensor will open up new opportunities in metastatic cancer status assessment in real-time and enhance personalized treatment.

## Data Availability

The data used to support the findings of this study are included within the article.

## Supplementary Materials

The following are available online at https://www.mdpi.com/2072-6694/13/4/873/s1, Figure S1: Cluster formation percentage in different cell concentrations, Figure S2: Cancer cell viability before and after culture, Figure S3: Viability of *C. elegans* before and after experimentation, Figure S4: Cluster area of cultures at various cell seeding concentrations. Table S1: Cancer detection technologies.

## Abbreviations

T	Tumor
N	Node
M	Metastasis
WB	Worm-based
C. elegans	Caenorhabditis elegans
CI	Chemotaxis index
PDMS	Polydimethylsiloxane
PET	Positron emission tomography
SPECT	Single-photon emission computer tomography
CTCs	Circulating tumor cells
MRI	Magnetic resonance imaging
CT scan	Computed tomography scan
T	Time
RBC	Red blood cells
