# Supplementary material for: Worm-Based Microfluidic Biosensor for Real-Time Assessment of the Metastatic Status
Source: Cancers (Basel). 2021 Feb 19;13(4):873. doi: 10.3390/cancers13040873 (PMC7922733; doi:10.3390/cancers13040873)
Supplement: Supplementary file 1 [file cancers-13-00873-s001.pdf]

cancers

MDPI

Communication

Supplementary Materials: Worm-Based Microfluidic Biosensor for Real-Time Assessment of the Metastatic Status

Jing Zhang, Song Lin Chua and Bee Luan Khoo

Supplementary Table

Supplementary Table S1. Cancer detection technologies

Technology		*Advantages*	*Limitations*	*Ref*
Biomarkers	PSA	Non-invasive<br>Organ-specific	Sensitivity (67.5-80%)<br>Low in specificity	[1,2]
	CA125	Non-invasive	Sensitivity (< 80%)<br>Low in specificity	[1,3]
	CEA	Non-invasive	Sensitivity (~80%)<br>Low in specificity (~70%)	[4]
Imaging	MRI	High contrast and spatial resolution<br>High sensitivity (~90%)<br>Non-invasive	Unable to detect lymph node metastases<br>High costs	[5–7]
	PET	Applicable for lymph node metastases<br>Non-invasive	Signal overlap<br>Affected by patient conditions such as blood glucose or psychotropic drugs	[8–10]
Histopathology	H&E	Standardized morphological identification<br>Long-term preservation	Invasive<br>Specific types of cells<br>Time-consuming	[11,12]
Chemotactic agents	WB biosensor	Inexpensive<br>Non-invasive<br>Ease of operation	Not specific to cancer subtypes or organs	

Supplementary Figures

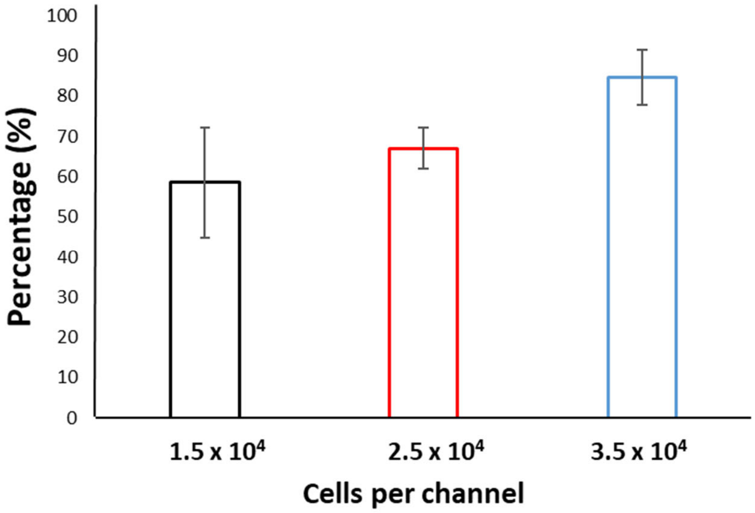

Supplementary Figure S1. Cluster formation percentage in different cell concentrations. The cluster formation percentage is the highest at 3.5×10^4 cells per channel.

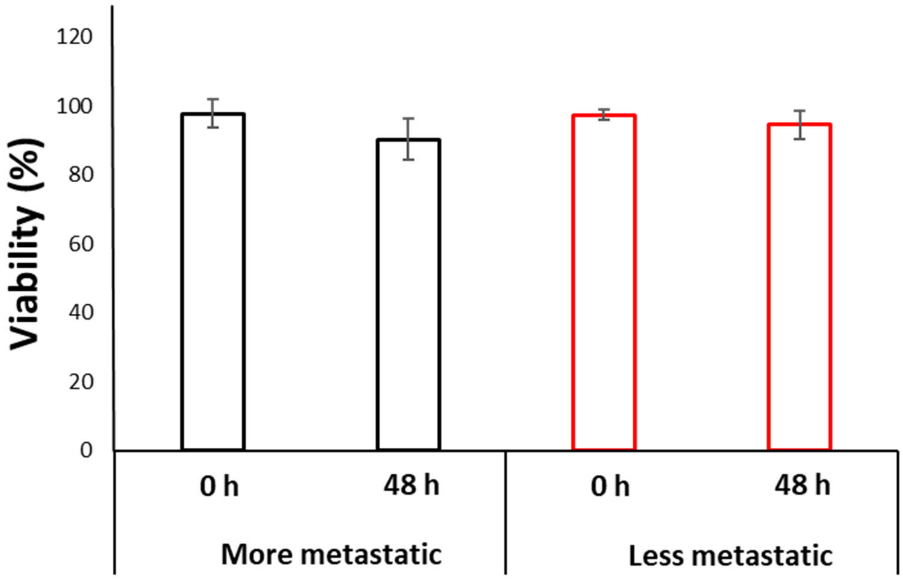

Supplementary Figure S2. Cancer cell viability before and after culture. Viability of cancer cells of the more metastatic phenotype (MDA-MB-231) and cancer cells of the less metastatic phenotype (MCF-7) before (0 h) and after sample collection (48 h). No significant differences between the time points were observed.

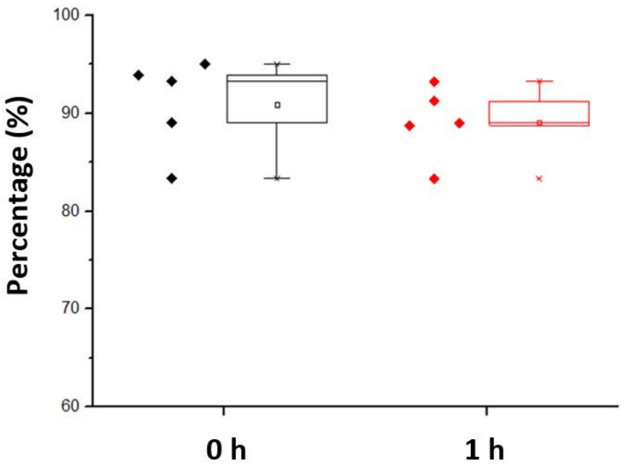

Supplementary Figure S3. Viability of *C. elegans* before and after experimentation. Box plot demonstrating the viability of *C. elegans* before (0 h) and after (1 h) experiments. No significant differences were observed.

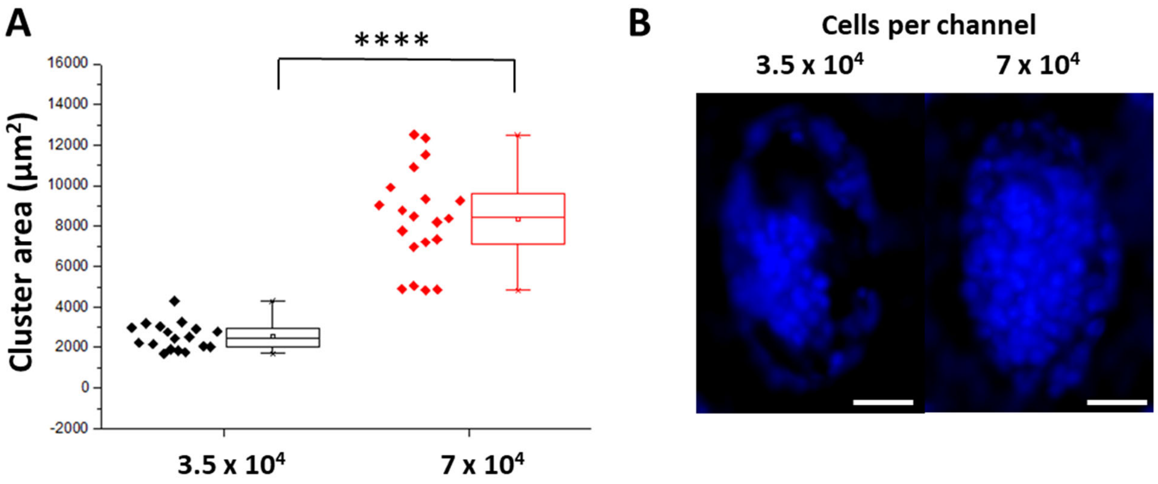

Supplementary Figure S4. Cluster area of cultures at various cell seeding concentrations. A) Box plot demonstrating the corresponding cluster area obtained with seeding concentrations of 3.5 × 10^4 and 7 × 10^4 cells per channel after 48 h. B) Representative images of clusters obtained with seeding concentrations of 3.5 × 10^4 and 7 × 10^4 cells per channel after 48 h. Cell nuclei were labeled with Hoechst dye. **** states for $p < 0.00001$. Scale bar 50 µm.

References

1. Meany, D.L.; Sokoll, L.J.; Chan, D.W. Early detection of cancer: immunoassays for plasma tumor markers. *Expert Opin. Med. Diagn.* **2009**, *3*, 597–605.
2. Barry, M.J. Prostate-specific–antigen testing for early diagnosis of prostate cancer. *N. Engl. J. Med.* **2001**, *344*, 1373–1377.
3. Duffy, M.J.; Bonfrer, J.M.; Kulpa, J.; Rustin, G.J.S.; Soletormos, G.; Torre, G.C.; Tuxen, M.K.; Zwirner, M. CA125 in ovarian cancer: European Group on Tumor Markers guidelines for clinical use. *Int. J. Gynecol. Cancer* **2005**, *15, 679–691.*
4. Duffy, M.J. Carcinoembryonic antigen as a marker for colorectal cancer: is it clinically useful? *Clin. Chem.* **2001**, *47*, 624–630.
5. Di Gioia, D.; Stieber, P.; Schmidt, G.; Nagel, D.; Heinemann, V.; Baur-Melnyk, A. Early detection of metastatic disease in asymptomatic breast cancer patients with whole-body imaging and defined tumour marker increase. *Br. J. Cancer* **2015**, *112*, 809–818.
6. Engelhard, K.; Hollenbach, H.P.; Wohlfart, K.; Von Imhoff, E.; Fellner, F.A. Comparison of whole-body MRI with automatic moving table technique and bone scintigraphy for screening for bone metastases in patients with breast cancer. *Eur. RadioL.* **2004**, *14*, 99–105.
7. Schmidt, G.; Dinter, D.; Reiser, M.F.; Schoenberg, S.O. The uses and limitations of whole-body magnetic resonance imaging. *Dtsch. Ärztebl. Int.* **2010**, *107*, 383–389.
8. Lind, P.; Igerc, I.; Beyer, T.; Reinprecht, P.; Hausegger, K. Advantages and limitations of FDG PET in the follow-up of breast cancer. *Eur. J. Nucl. Med. Mol. Imaging* **2004**, *31*, S125–S134.
9. Kato, T.; Tsukamoto, E.; Kuge, Y.; Takei, T.; Shiga, T.; Shinohara, N.; Katoh, C.; Nakada, K.; Tamaki, N. Accumulation of [11 C] acetate in normal prostate and benign prostatic hyperplasia: comparison with prostate cancer. *Eur. J. Nucl. Med. Mol. Imaging* **2002**, *29*, 1492–1495.
10. Salmon, E.; Ir, C.B.; Hustinx, R. Pitfalls and limitations of PET/CT in brain imaging. *Semin. Nucl. Med.* **2015**, *45*, 541–551.
11. Alturkistani, H.A.; Tashkandi, F.M.; Mohammedsaleh, Z.M. Histological stains: a literature review and case study. *Glob. J. Health Sci.* **2016**, *8*, 72–79.
12. Fischer, A.H.; Jacobson, K.A.; Rose, J.; Zeller, R. Hematoxylin and eosin staining of tissue and cell sections. *CSH Protoc.* **2008**, doi:10.1101/pdb.prot4986.
